# Mannan-Binding Lectin in Diabetic Kidney Disease: The Impact of Mouse Genetics in a Type 1 Diabetes Model

**DOI:** 10.1155/2012/678381

**Published:** 2012-05-08

**Authors:** Jakob Appel Østergaard, Mette Bjerre, Satish Posettihalli RamachandraRao, Kumar Sharma, Jens Randel Nyengaard, Troels Krarup Hansen, Steffen Thiel, Allan Flyvbjerg

**Affiliations:** ^1^Department of Endocrinology and Internal Medicine, Aarhus University Hospital and The Medical Research Laboratories, Institute of Clinical Medicine, Faculty of Health Sciences, Aarhus University, 8000 Aarhus C, Denmark; ^2^Center for Renal Translational Medicine, University of California, San Diego/VA Medical System, La Jolla, CA 92093-0711, USA; ^3^Stereology and Electron Microscopy Laboratory, Centre for Stochastic Geometry and Advanced Bioimaging, Aarhus University Hospital, 8000 Aarhus C, Denmark; ^4^Department of Medical Microbiology and Immunology, Faculty of Health Sciences, Aarhus University, 8000 Aarhus C, Denmark

## Abstract

*Background*. Mannan-binding lectin (MBL) is involved in the development of diabetic nephropathy. MBL is a part of the innate immune system where it can activate the complement system. Serum MBL level predicts later renal impairment in diabetes patients. Direct involvement of MBL in the development of diabetic kidney disease is observed in one animal strain. However, this involvement may differ among the animal strains. We thus examined the impact of the genetic background on the role of MBL in diabetic nephropathy. *Materials/Methods*. C57BL/6JBomTac and 129S6/SvEvTac mice were compared. In both strains, experimental type 1 diabetes was induced in wild-type (WT) and MBL-knockout (MBL-KO) mice by streptozotocin. Nondiabetic WT and MBL-KO mice were used as controls. We tested if MBL modified the diabetes-induced kidney changes by two-way ANOVA allowing for interaction. *Results*. MBL aggravated diabetes-induced kidney growth and glomerulus enlargement in C57BL/6JBomTac mice. MBL did not modify diabetes effects on glomerular basement membrane thickness or mesangial volume in any strain. Diabetes-induced changes in renal gene transcription of growth factors and matrix components were unaffected by MBL. *Conclusions*. Strain-specific MBL effects were found on downstream diabetic kidney changes. This emphasizes the importance of genetic background in this model of diabetic complications.

## 1. Introduction

Diabetic nephropathy is the most common cause of end-stage renal disease in many Western countries [1–5]. The cumulative incidence of diabetic nephropathy is about one-third in different diabetic populations [6–10]. Although the incidence has fallen during the last decades, better treatment and identification of high-risk patients are still needed [11]. An unknown genetic predisposition to diabetic vascular complications is plausible since not all patients at risk will develop diabetic nephropathy and because diabetic nephropathy clusters in families [12, 13].

The complement system is an inflammation activating part of the innate immune system and appears to be important for vascular damage in the heart and in the kidneys of diabetic patients [14]. Mannan-binding lectin (MBL) is a recognition molecule and the first step of the lectin pathway of complement activation. Interindividual variations in MBL serum level may account for some predisposition to vascular late complications to diabetes. The association between high MBL and renal dysfunction is well-described in type 1 diabetes [15–17]. Furthermore, the MBL level in the early stage of type 1 diabetes predicts the risk of future renal dysfunction [18]. In type 2 diabetes, the association between MBL and renal function is less documented. The MBL level tends to predict renal impairment in type 2 diabetes and combined with C-reactive protein the prognostic value of MBL level reaches statistical significance [19]. We have previously studied the effects of MBL on early diabetic kidney disease by MBL-KO mice backcrossed for six generations on the C57BL/6JBomTac background [20]. In the present study we examined the role of MBL in animals backcrossed for 12 generations which ensured a more homogeneous group of animals. MBL interaction with effects of diabetes may differ between the models, for example, between genetic backgrounds. As the C57BL/6JBomTac strain is relatively more resistant to diabetic kidney changes than the 129S6/SvEvTac strain, we compared these strains in the present study [21]. In this setup, we evaluated the genetic impact on kidney changes after the induction of experimental type 1 diabetes. Nondiabetic WT and nondiabetic MBL-KO animals were used as controls.

## 2. Methods

### 2.1. Animals

We used female C57BL/6JBomTac and female 129S6/SvEvTac mice. Both WT and MBL-KO mice were included in both strains (MBL-KO mice lack both murine forms of MBL, MBL-A, and MBL-C). The development of MBL-KO animals have previously been described [22]. In brief, MBL double-knockout mice were developed on a mixed C57BL/6 × Sv129EvSv background and later backcrossed onto both C57BL/6JBomTac and to 129S6/SvEvTac background. WT animals of both strains were purchased from Taconic (Ry, Denmark). MBL-KO animals came from our own breeding (backcrossed 12 generations or more on C57BL/6JBomTac or 129S6/SvEvTac, resp.). At study start, C57BL/6JBomTac animals were 10 weeks old and 129S6/SvEvTac animals were 11 weeks old. Diabetes was induced by streptozotocin (STZ) (Sigma-Aldrich Corp., St. Louis, MO, USA) as described below. All C57BL/6JBomTac animals were sacrificed 12 weeks after diabetes induction and all 129S6/SvEvTac animals were sacrificed 14 weeks after diabetes induction. The varying diabetes duration is explained by different rates of responses to STZ (6 weeks in C57BL/6JBomTac animals and 4 weeks in 129S6/SvEvTac animals) and coincident time of sacrifice. The animals were housed 5 to 7 animals per cage in a room with a 12 : 12-hour artificial light cycle (light 7.00 h to 19.00 h.), a temperature of 21±1°C, and a humidity of 55±5%. The animals had free access to standard chow (Altromin #1324; Lage, Germany) and tap water throughout the experiment. Food consumption was determined at group basis at the end of the study. Blood glucose was measured in tail-capillary blood as described below. Body weight and blood glucose were measured weekly throughout the study. Animals with signs of illness (*n* = 2, please see section 2.2), ketonuria (*n* = 0), and more than 10% weight loss (*n* = 0) were excluded. The study complied with the Danish regulations for care and use of laboratory animals.

### 2.2. Design

Four groups were made from both strains; diabetes (yes/no) and MBL-KO (yes/no). In this manner C57BL/6JBomTac animals were randomly divided into four groups: (1) control WT (*n* = 7), (2) diabetic WT (*n* = 7), (3) control MBL-KO (*n* = 6), and (4) diabetic MBL-KO (*n* = 6). Likewise, 129S6/SvEvTac mice were randomly divided into four groups: (1) control WT (*n* = 5), (2) diabetic WT (*n* = 6), (3) control MBL-KO (*n* = 5), and (4) diabetic MBL-KO (*n* = 6).

Two C57BL/6JBomTac animals were excluded: one from the control MBL-KO group because of hydronephrosis and one from the diabetic MBL-KO group because of severe polycystic kidney disease. Serum from one 129S6/SvEvTac control MBL-KO mouse was lost during preparation.

### 2.3. Induction of Diabetes

Diabetes was induced by five intraperitoneal STZ-injections on five consecutive days using doses of 45 mg/kg body weight. If not diabetic after these injections, the animals were re-injected intra-peritoneally with doses of 45 mg STZ/kg body weight until diabetes was achieved. Animals were considered as diabetic when blood glucose was above 18 mmol/L.

### 2.4. Determination of Blood Glucose and Urinalysis

Blood glucose was measured in tail-capillary blood by Contour (Bayer Diabetes Care, Kongens Lyngby, Denmark). Urine was tested for glucose and ketone bodies by Combur^5^ Test D (Roche Diagnostics GmbH, Mannheim, Germany).

### 2.5. Sacrifice and Samples for Examination

At the end of the study, urine was collected in metabolic cages individually and stored at −20°C until assayed. At sacrifice, the animals were anesthetized by an intraperitoneal dose of 0.5 mg/g body weight ketamine and 0.2 mg/g body weight xylazine (Ketaminol Vet and Narcoxyl Vet, resp., Intervet, Skovlunde, Denmark). Nonfasting blood samples were drawn in heparinized capillary tubes from the retroorbital venous plexus, and serum was separated. Serum samples were stored at −20°C until analyzed. Kidneys, liver, and heart from all animals were dissected and weighed. The poles and the middle pieces of the right kidneys were snap-frozen in liquid nitrogen for later mRNA analyses and stored at −80°C. Left kidney was fixed in a mixture of 3% formaldehyde and 1% glutaraldehyde, cut in 1 mm slices and 2 slices were randomly sampled. From these two slices, five random pieces were area-sampled and embedded in Epon 825 for electron microscopy, whereas the remaining tissue was embedded in paraffin for light microscopy. Morphological methods are described below.

### 2.6. Determination of Urinary Albumin Excretion (UAE) and Creatinine Clearance

Albumin was determined in urine collections by Mouse Albumin ELISA Quantification Kit (Bethyl Laboratories, Inc., Montgomery, TX, USA) according to the manufacturer's instructions. Creatinine was measured by high-performance liquid chromatography as previously described [23].

### 2.7. Estimation of Glomerular Volume

The kidney slices embedded in paraffin were cut in 15 *μ*m thick sections on a rotatory microtome and stained with Periodic acid-Schiff. In these sections, blinded estimation of glomerular volume fraction was performed by point counting (CAST software, Olympus, Copenhagen, Denmark). Glomerulus-to-kidney ratio was calculated from these estimates based on 50 or more glomeruli per section.

### 2.8. Estimation of Glomerular Basement Membrane Thickness, Mesangial Fraction and Total Mesangial Volume

These estimates were examined by electron microscopy as previously described [20]. In brief, the five small pieces embedded in Epon 825 were cut in ultrathin sections on an RMC MT-7000 Ultramicrotome (Boeckeler Instruments Inc., AZ, USA). Electron microscopy images (CM10, Phillips, Eindhoven, Holland) were recorded with a Kodak megaplus 1.6i digital camera applying a multiple-Image alignment system onto a monitor.

### 2.9. Quantitative RT-PCR

The quantifications by RT-PCR were performed as previously described [20]. In brief, total cellular RNA was extracted from renal cortical tissue by a 6100 Nucleic Acid PrepStation (Applied Biosystems, Foster City, CA, USA). The quality of ribosomal RNA was estimated by agarose gel. Reverse transcription from RNA to DNA was performed with a Multiscribe Reverse Transcriptase kit (Applied Biosystems). The polymerase chain reaction was performed in triplicates of each sample in wells containing RNA, TaqMan Universal PCR MasterMix and amplifying primers purchased from Applied Biosystems. Ribosomal 18S was used as housekeeping gene. Liver RNA was used as negative controls. Data were analyzed with the ABI Prism 7000 Sequence Detector Software from Applied Biosystems. The relative quantification of target gene was calculated as described in the Users Bulletin 2, 1997 from Perkin-Elmer (Perkin-Elmer Cetus, Norwalk, CT, USA) [24].

### 2.10. Statistical Analysis

The study was designed as a 2 times 2 factorial experiment and thus analyzed by two-way ANOVA allowing for interaction. First, we tested if the diabetes effect was modified by MBL (effect modification or interaction). If interaction between diabetes and MBL was found, we tested the hypothesis that the two nondiabetic groups did not differ. Only if we could accept the hypothesis of no interaction, we could test the hypothesis that no isolated effect of MBL and diabetes could be found. The Kruskal-Wallis equality of population rank test was used if normality and equal variances could not be achieved by transformation and these data are therefore not evaluated for interaction, isolated MBL effect, or diabetic effect. All RT-PCR measurements were transformed by the natural logarithm for comparisons as ratios rather than differences. Unless otherwise stated, all data are expressed as mean (95% confidence interval), number as stated above in, except when other numbers are given. *P* < 0.05 was considered significant. Statistical analyses were performed by STATA 11.0 for Microsoft Windows.

## 3. Results

### 3.1. Animal Characteristics

Body weights of the animals are presented in Table 1. To best describe the overall level throughout this study, the diabetic groups were compared estimating blood glucose as area under the curve (days times glucose concentration). In both strains, diabetic animals had equal levels of blood glucose (Table 1). Glucose levels at start and end are listed in Table 1.

### 3.2. Kidney Morphology

**Figure 1:**
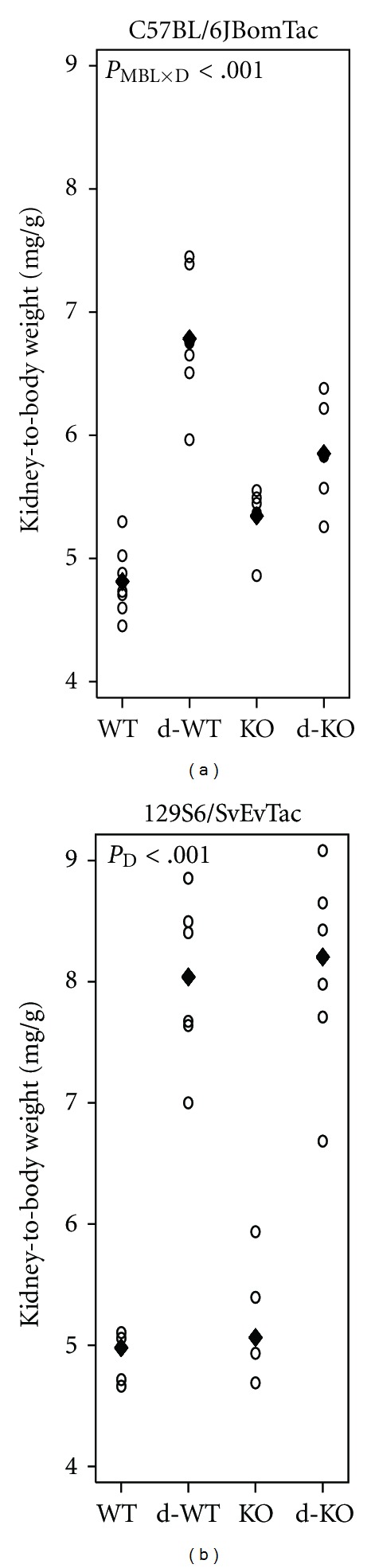
Kidney-to-body weight ratio. Kidney-to-body weight ratio by mouse strain: WT, diabetes WT (d-WT), MBL-KO (KO), and diabetes MBL-KO (d-KO). (a) C57BL/6JBomTac animals with mean indicated by “♦.” (b) 129S6/SvEvTac animals with median indicated by “♦” (median presented as untransformed data showed unequal variances among groups). Test statistics are indicated in plot area when significant. *P*_MBL×D_: interaction between MBL and diabetic effects; *P*_D_: diabetic change.

The diabetes-induced kidney hypertrophy significantly depended on MBL in C57BL/6JBomTac animals (interaction between diabetes and MBL, *P* < 0.001). Diabetes induced a 29% (15;45) larger increase in kidney-to-body weight ratio in WT animals (41% increase) compared with MBL-KO animals (9% increase) (Figure 1(a)). Kidney-to-body weight ratio was 11% (2;21) larger in nondiabetes WT animals compared with nondiabetic MBL-KO animals (*P* = 0.02). In 129S6/SvEvTac animals, no interaction was found and we therefore continued testing for an isolated difference between MBL-KO and WT animals and between diabetes and control animals (please refer to Methods Section for further details). Accordingly, we found that diabetic animals had larger kidney-to-body weight ratio than the control animals (59% (47;72), *P* < 0.001). MBL deficiency had no effect on kidney weight (Figure 1(b)).

**Figure 2:**
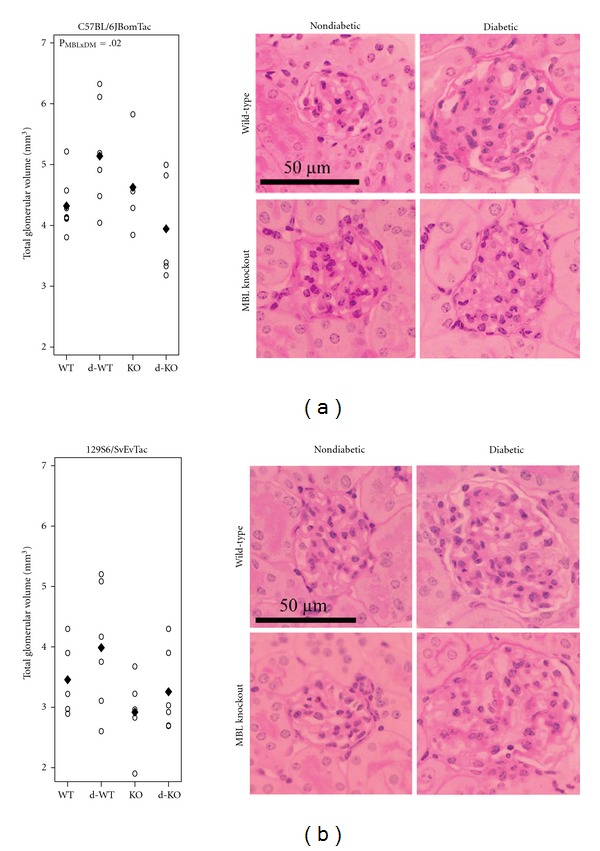
Glomerular volume. Total glomerular volume by mouse strain: WT, diabetes WT (d-WT), MBL-KO (KO), and diabetes MBL-KO (d-KO) and representative images from each group (total magnification 1855x). (a) C57BL/6JBomTac animals with mean indicated by “♦.” (b) 129S6/SvEvTac animals with mean indicated by “♦.” Test statistics are indicated in plot area when significant. *P*_MBL×D_: interaction between MBL and diabetic effects.

MBL modified the diabetes-induced glomerulus enlargement in C57BL/6JBomTac animals (Figure 2(a)). Due to diabetes, total glomerulus volume increased 40% (7;84) more in WT animals compared to MBL-KO animals (*P* = 0.02). No differences were seen in the control WT and control MBL-KO groups. In 129S6/SvEvTac animals no signs of interaction and no diabetic changes were seen, nor were any consequences of MBL knockout found. Basement membrane thickness and mesangium-to-glomerulus volume fraction did not differ among groups in both strains (data not shown).

### 3.3. UAE and Creatinine Clearance

**Figure 3:**
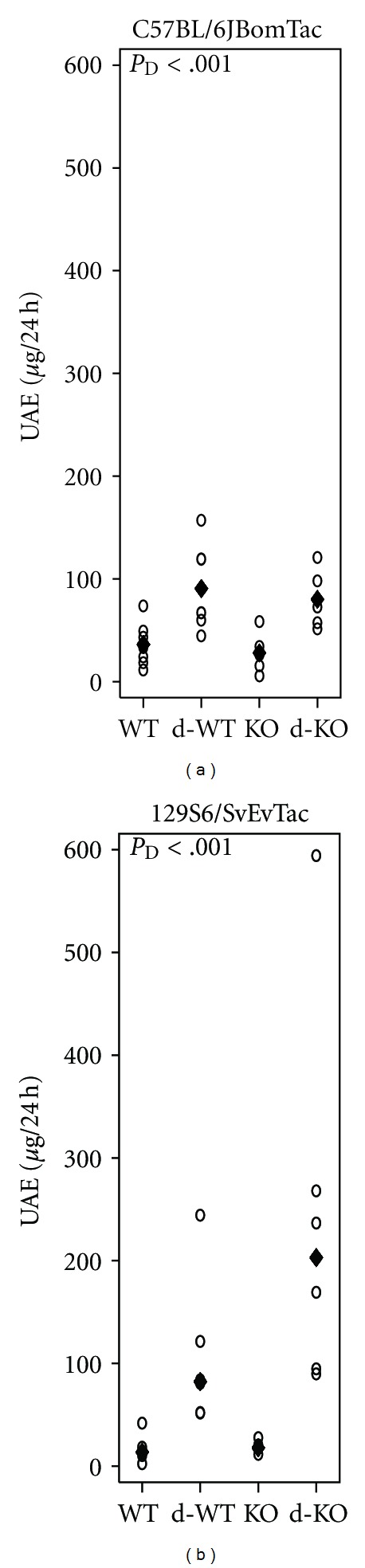
Urinary albumin excretion. Urinary albumin excretion (UAE) by mouse strain: WT, diabetes WT (d-WT), MBL-KO (KO), and diabetes MBL-KO (d-KO). (a) C57BL/6JBomTac animals with mean indicated by “♦.” (b) 129S6/SvEvTac animals with “♦” indicating the median (median presented as untransformed data showed unequal variances among groups). Test statistics are indicated in plot area when significant. *P*_D_: diabetic change.

The diabetes-induced increase in UAE was not modified by MBL in any of the strains. Diabetic C57BL/6JBomTac animals showed a 3.0-fold (1.8;5.0) increase in UAE compared with nondiabetic animals (*P* < 0.001) (Figure 3(a)). Diabetes induced an 8.9-fold increase in UAE in 129S6/SvEvTac animals compared with nondiabetic controls (4.6;17.4), *P* < 0.001. In both strains there were no effect of MBL deficiency (Figure 3(b)).

**Figure 4:**
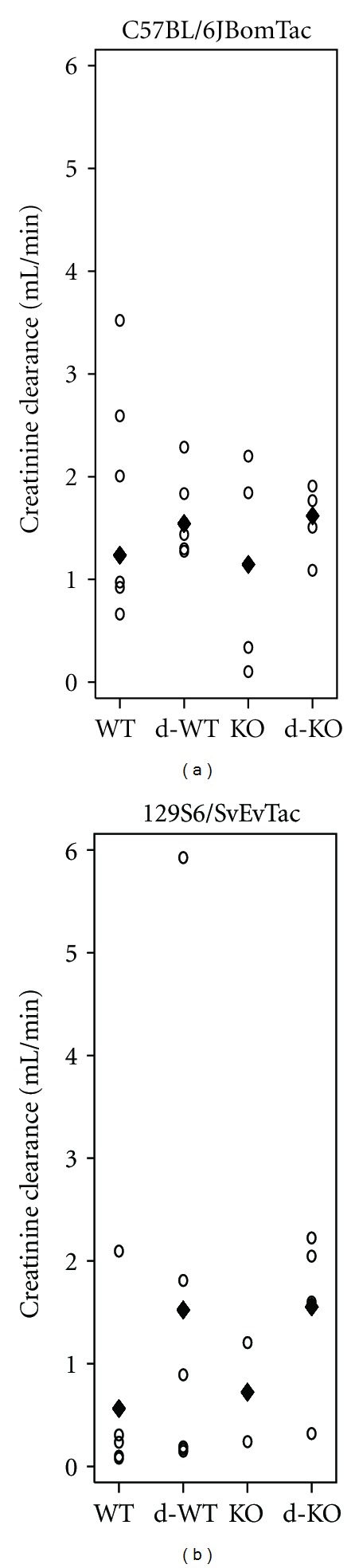
Creatinine clearance. Creatinine clearance by mouse strain: WT, diabetes WT (d-WT), MBL-KO (KO), and diabetes MBL-KO (d-KO). (a) C57BL/6JBomTac animals with median indicated by “♦” (median presented as data could not be fitted to normality). (b) 129S6/SvEvTac animals with “♦” indicating the mean. In 129S6/SvEvTac animals, creatinine clearance could not be estimated in two animals because of technical issues (both control MBL-KO animals) and in two animals because of lost serum (one control MBL-KO animal and one diabetic MBL-KO animal). No statistical significance was found on interaction between MBL and diabetic effects, or on diabetic change, or on impact of MBL deficiency.

Creatinine clearance did not differ among groups in C57BL/6JBomTac animals (Figure 4(a)). In the 129S6/SvEvTac animals, no effectmodication between diabetes and MBL was found on creatinine clearance and creatinine clearance was not altered in MBL-deficient animals or in diabetes (Figure 4(b)).

### 3.4. Renal Gene Transcription

Gene transcripts involved in diabetic kidney changes were measured by RT-PCR (Table 2). MBL did not interact with the diabetes-induced changes on the tested gene transcripts. In both strains, transcription of growth factors important for sclerotic changes was upregulated in diabetes, that is, transforming growth factor *β* and connective tissue growth factor. Genes for matrix proteins were also transcribed at higher levels in diabetes (i.e., fibronectin and collagen IV*α*1), whereas vascular endothelial growth factor-A mRNA expression was downregulated in both strains.

Connective tissue growth factor, fibronectin and vascular endothelial growth factor receptor 2 transcriptions were downregulated in MBL-KO animals compared with WT in C57BL/6JBomTac animals. In 129S6/SvEvTac MBL-KO animals, transcriptions of transforming growth factor *β*, collagen IV*α*1, and vascular endothelial growth factor receptor 2 were upregulated compared with WT animals.

## 4. Discussion

In the present study causal involvement of MBL in the development of key characteristic diabetic kidney changes were demonstrated. We found that growth of kidneys and glomeruli in diabetes were significantly modified by MBL in agreement with previous findings in MBL-KO animals backcrossed for only six generations [20]. As hypothesized, the role of MBL was dependent on the genetic background of the mice. This indicates an important genetically determined difference in susceptibility to MBL regarding the development of diabetic kidney disease. Previous studies characterized diabetic kidney changes in various mouse models and found great influence of the genetic background [25, 26]. The ability to mimic the different characteristics of human diabetic nephropathy varies between models [21]. Accordingly, STZ diabetes in C57BL/6JBomTac animals, and to some extent also in 129S6/SvEvTac animals, imitates the structural kidney changes in human diabetes better than functional alternations in UAE [25, 26]. In the present report we find that STZ diabetes induces kidney changes in accordance with diabetic nephropathy in both strains. The models limitations to resemble functional diabetic kidney changes may account for the inability to find the association between MBL and diabetes-induced elevated UAE, which has been reported in numerous human studies [15, 17–19, 27, 28]. In the present study, also a higher kidney-to-body weight ratio was found in control C57BL/6JBomTac MBL-KO animals compared with control WT animals. Different growth rates in KO compared with WT animals may account for part of this. We have previously shown a trend of MBL to modify the diabetes-induced increases in glomerulus basement membrane thickness and mesangial volume [20]. However, this could not be repeated in the present study, most likely due to longer diabetes duration and to the more genetically similar group with animals backcrossed for more than 12 generations. Diabetes-induced changes in renal gene transcription of known key mediators were not found to be modified by MBL in this study. The time of determination might influence the outcome of these measurements. We found a decreased vascular endothelial growth factor A transcription in both mouse strains at an early stage of diabetic kidney disease. Differing reports have been published on vascular endothelial growth factor levels. Though most studies find an upregulation of vascular endothelial growth factor, we have consistently found downregulation of vascular endothelial growth factor A in STZ-diabetic mice after diabetes durations of 8 to 12 weeks [29–32]. Differing sample site and diabetes duration may explain these observations.

The importance of genetic composition regarding susceptibility to diabetic nephropathy is illustrated from observations in twins. Seaquist et al. described a strong association between genetic composition and risk of end-stage renal disease in type 1 diabetes [13]. In accordance with this observation, the MBL genotype is found to be associated with risk of diabetic nephropathy, which may possibly explain parts of the genetic predisposition [15, 18]. Still, Kaunisto and colleagues did not find the same association in a Finnish population [16].

Speculations on alternative pathways of interaction between MBL and diabetic kidney changes are however unavoidable. MBL binding to human cells has been demonstrated by flow cytometry [33]. Increasing evidence of complement attack on self-cells in the diabetic milieu has been reported, and, recently, MBL binding to glycated protein and initiation of the complement system have been demonstrated [34]. Dysfunction of complement regulation in diabetes has also been suggested. Acosta et al. and Qin et al. showed glycation-mediated inhibition of complement regulatory protein CD59 as a possible mechanism of growth factor release in diabetes [35, 36]. Detrimental effects of the complement system are known from studies of ischemia-reperfusion injury, and inhibition of terminal complement activation reduces infarct size in experimental settings [37]. More recently, a key role of MBL in ischemia-reperfusion injury in different organs, including heart and kidney, has been reported [38, 39]. In these situations, generation of neoepitopes on cells during ischemia may initiate complement activation. In conclusion, this study presents data illustrating an important effect of MBL on key downstream hallmarks of diabetic kidney disease. This effect was only observed in one of the two studied mouse strains indicating that the genetic background is important. However, examination at several different diabetes durations should be made to be able to compare with the effects on early diabetic kidney changes observed in the present study. By measurement of renal gene expression, we also find that the classic up-stream mediators of these changes are unaffected by MBL-KO. To what extent this is caused by inability to measure renal gene expression at the right time of disease development is not known. Alternatively, the existence of undiscovered pathways linking complement and diabetic kidney disease are possible. New studies should explore this evident connection between MBL and vascular complications to diabetes.

## Figures and Tables

**Table 1:** Body weight and blood glucose.

	Group					ANOVA		
	Strain	WT	d-WT	KO	d-KO	*P*_MBL×D_	*P*_D_	*P*_MBL Knockout_
Body weight (g)	C57BL/6JBomTac, start	21.1 (19.8;22.5)	20.8 (19.4;22.2)	19.6 (18.0;21.2)	20.7 (19.1;22.3)	NS	NS	NS
	C57BL/6JBomTac, end	25.3 (24.2;26.4)	22.4 (21.3;23.5)	25.3 (24.0;26.6)	22.2 (20.9;23.5)	NS	<0.001	NS
	129S6/SvEvTac, start	20.9 (19.5;22.6)	20.6 (19.4;21.8)	18.2 (16.8;19.5)	17.7 (16.5;19.0)	NS	NS	<0.001
	129S6/SvEvTac, end	25.7 (23.7;27.7)	23.7 (21.8;25.5)	22.4 (20.3;24.4)	20.8 (18.9;22.6)	NS	0.06	0.003

						Student's *t-*test		
						*P*_WT versus KO_	*P*_d-WT versus d-KO_	

Blood glucose (mmol/L)	C57BL/6JBomTac, start	6.3 (5.4;7.3)	5.9 (4.9;6.8)	6.1 (4.9;7.2)	7.5 (6.4;8.7)	NS	0.03	
	C57BL/6JBomTac, end	6.0 (2.8;9.2)	20.7 (17.5;23.9)	6.2 (2.4;10.0)	22.0 (18.2;25.7)	NS	NS	
	C57BL/6JBomTac, area under the curve						NS	
	129S6/SvEvTac, start	6.2 (5.3;7.0)	6.8 (6.1;7.6)	6.3 (5.5;7.1)	7.2 (6.4;7.9)	NS	NS	
	129S6/SvEvTac, end	6.2 (4.3;8.2)	20.4 (18.6;22.2)	6.4 (4.5;8.3)	16.8 (15.0;18.6)	NS	0.007	
	129S6/SvEvTac, area under the curve						NS	

Body weight and blood glucose by strain at start and end of study in wild-type (WT), diabetes WT (d-WT), MBL knockout (KO), and diabetes KO (d-KO).

Values indicate mean (95% confidence interval).

Comparisons were made with ANOVA (body weight) and Student's *t*-test (blood glucose).

*P*_MBL×D_: interaction between diabetes and MBL.

*P*_D_: diabetic versus nondiabetic animals.

*P*_MBL_: MBL knockout versus WT animals.

*P*_WT versus KO_: wild-type versus knockout.

*P*_d-WT versus d-KO_: diabetic wild-type versus diabetic knockout; NS: *P* > 0.05.

**Table 2:** Renal gene expression.

	ANOVA		
mRNA, strain	*P*_MBL×D_	*P*_D_	*P*_MBL knockout_
TGF-*β*, C57BL/6JBomTac	NS	2.3-fold (1.7;3.1), *P* < 0.001	NS
TGF-*β*, 129S6/SvEvTac	NS	2.5-fold (1.8;3.6), *P* < 0.001	44% (2;104), *P* = 0.04
CTGF, C57BL/6JBomTac	NS	33% (9;64), *P* < 0.008	−34% (−19;−46), *P* < 0.001
CTGF, 129S6/SvEvTac	NS	2.2-fold (1.5;3.1), *P* < 0.001	NS
Fibronectin, C57BL/6JBomTac	NS	2.6-fold (1.9;3.7), *P* < 0.001	−30% (−2;−50), *P* = 0.04
Fibronectin, 129S6/SvEvTac	NS	3.6-fold (2.4;5.5), *P* < 0.001	NS
Collagen IV*α*1, C57BL/6JBomTac	a	*P* < 0.03^b^	NS^*c*^
Collagen IV*α*1, 129S6/SvEvTac	NS	2.4-fold (2.1;2.8), *P* < 0.001	16% (0;34), *P* = 0.04
VEGF-A, C57BL/6JBomTac	NS	−51% (−22;−69), *P* = 0.004	NS
VEGF-A, 129S6/SvEvTac	NS	−24% (−6;−38), *P* = 0.02	NS
VEGFR-2, C57BL/6JBomTac	NS	NS	−30% (−14;−43), *P* = 0.002
VEGFR-2, 129S6/SvEvTac	NS	NS	41% (12;77), *P* = 0.005
Nephrin, C57BL/6JBomTac	NS	NS	NS
Nephrin, 129S6/SvEvTac	NS	NS	NS

Expression of mRNA measured by RT-PCR relative to 18S mRNA.

*P*_MBL×D_: interaction between diabetes and MBL.

*P*_D_: diabetic versus nondiabetic animals.

Values indicate diabetes compared with control (95% confidence interval). *P*_MBL_: MBL knockout versus WT animals. Values indicate MBL knockout compared with wild-type (95% confidence interval).

NS: *P* > 0.05.

^a^: values could not be fitted to normality and therefore Wilcoxon rank-sum tests were used to test effects of diabetes and MBL.

^b^: *P* = 0.025 (WT versus diabetic WT, by Wilcoxon rank-sum tests) and *P* = 0.028 (MBL-KO versus diabetic MBL-KO, by Wilcoxon rank-sum test).

^c^: WT versus MBL-KO by Wilcoxon rank-sum test.

One diabetic WT was omitted from analyses for technical reasons.
